# Supplementary figures and images for: Xenopus Hybrids Provide Insight Into Cell and Organism Size Control
Source: Front Physiol. 2018 Dec 4;9:1758. doi: 10.3389/fphys.2018.01758 (PMC6288844; doi:10.3389/fphys.2018.01758)

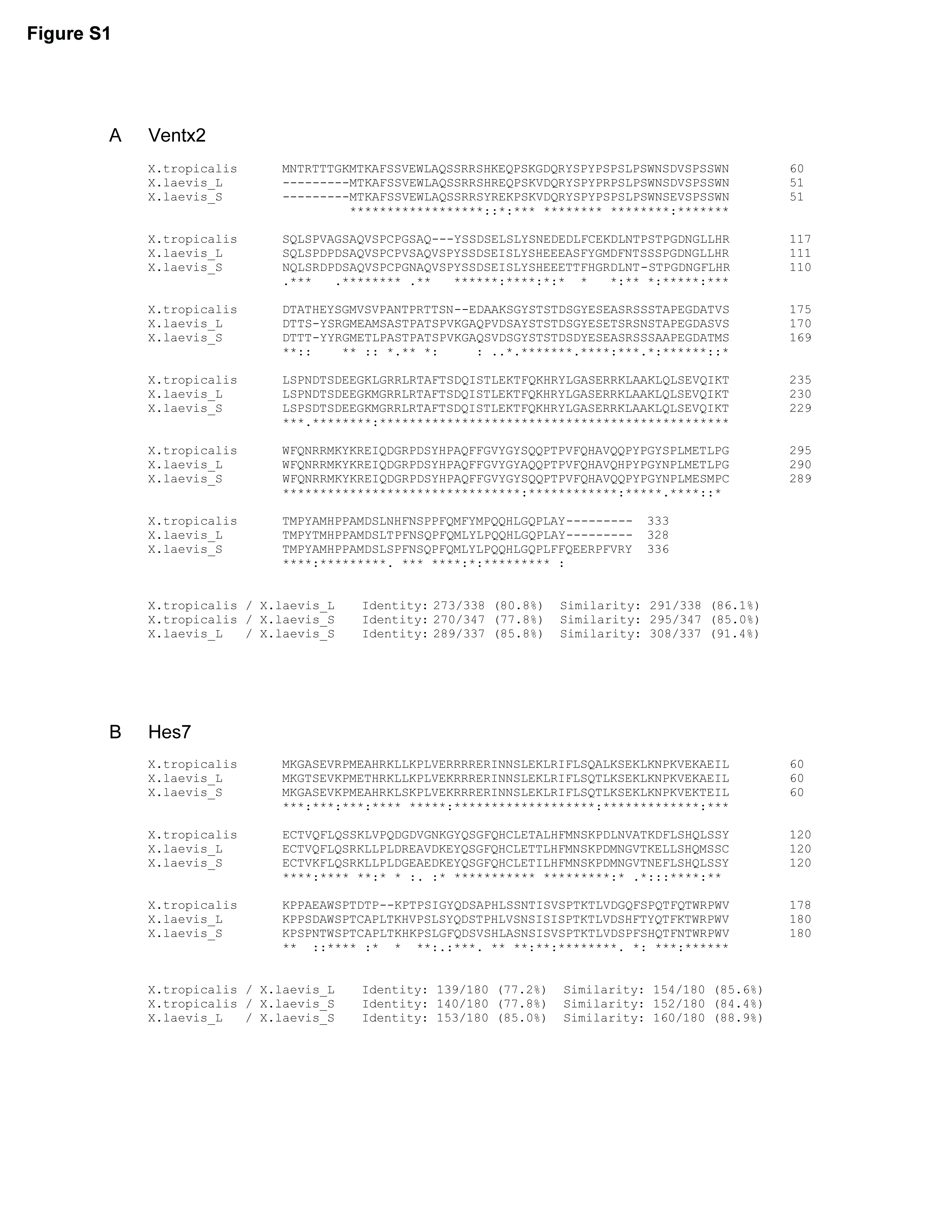

Supplement: Supplementary Figure S1 — Protein sequence alignments of Xenopus Ventx2 and Hes7. (A) Ventx2 protein sequences of X. tropicalis, X. laevis L, and S copies aligned using Clustal Omega. (B) Hes7 protein sequences of X. tropicalis, X. laevis L, and S copies aligned using Clustal Omega. For (A,B), identities and similarities were calculated using EMBOSS Needle. [file Image_1.TIF]

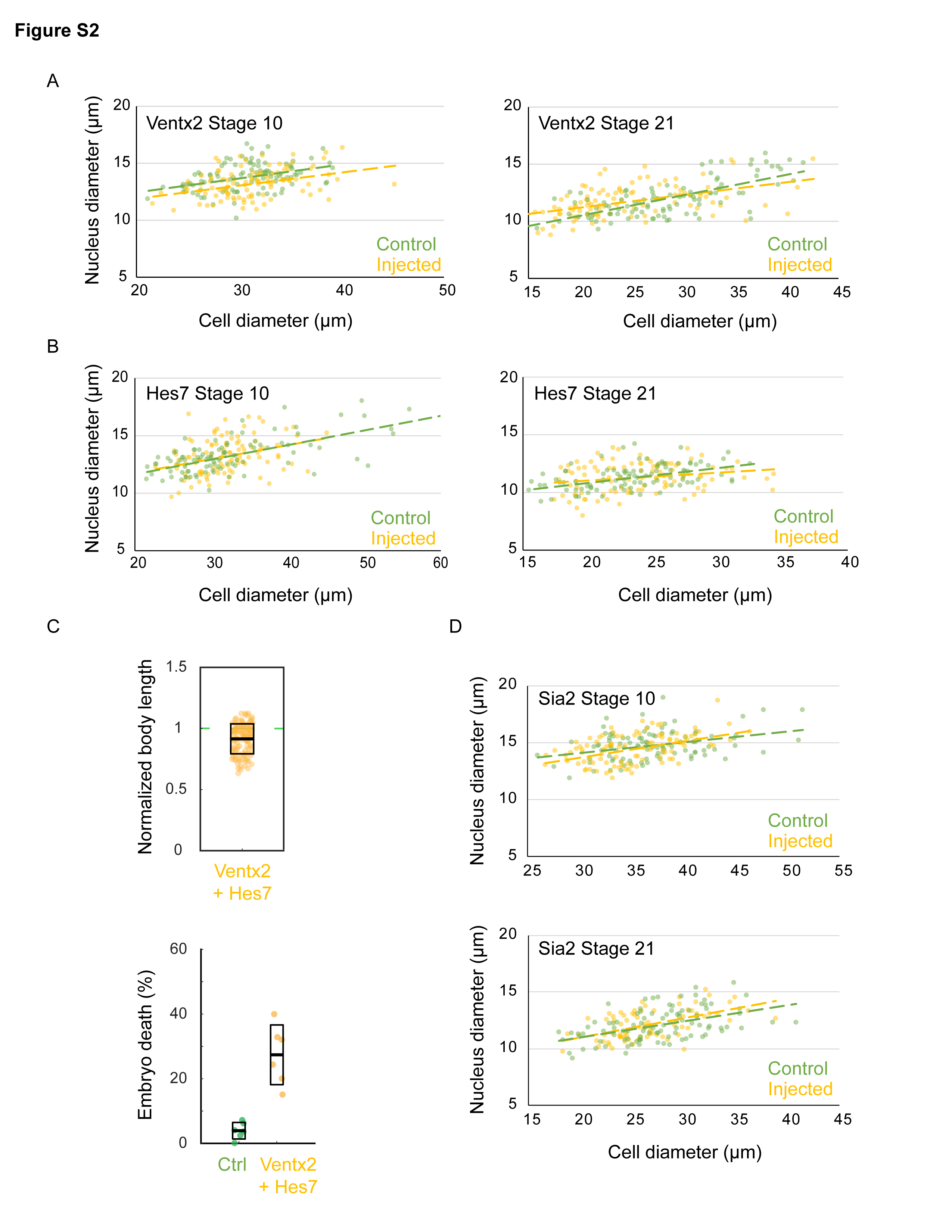

Supplement: Supplementary Figure S2 — Nuclear to cell size relationships post-zygotic genome activation in injected X. laevis embryos. (A) Nuclear diameter vs. cell diameter in Ventx2-injected X. laevis embryos at stage 10 and 21. Analysis of covariance at stage 10 gave a p-value of 0.865 and at stage 21 of 0.016. (B) Nuclear diameter vs. cell diameter in Hes7-injected X. laevis embryos at stage 10 and 21. Analysis of covariance at stage 10 gave a p-value of 0.820 and at stage 21 of 0.077. (C) Normalized body length of tailbud stage X. laevis embryos co-injected with Ventx2 and Hes7 (top). Percent fatality in Ventx2 and Hes7 co-injected embryos (bottom). Embryos were injected with 375 pg mRNA of each factor for a total injection of 750 pg. Thick line inside box = average length, upper, and lower box boundaries = ±SD. (D) Nuclear diameter vs. cell diameter in Sia2 injected X. laevis embryos at stages 10 and 21. Analysis of covariance at stage 10 gave a p-value of 0.171 and at stage 21 of 0.497. [file Image_2.tif]
